# It is time to get real when trying to predict educational performance

**DOI:** 10.7554/eLife.55720

**Published:** 2020-03-13

**Authors:** Cecile Janssens

**Affiliations:** Rollins School of Public Health, Emory UniversityAtlantaUnited States

**Keywords:** genetics, education, prediction, polygenic score, achievement, ALSPAC, Human

## Abstract

A study of 3,500 children in the UK shows that data on socioeconomic background and previous educational achievements can better predict how students will perform at school than genetic data.

**Related research article** Morris TT, Davies NM, Davey Smith G. 2020. Can education be personalised using pupils’ genetic data?. *eLife* **9**:e49962. doi: 10.7554/eLife.49962

Interest in using polygenic scores to make predictions is skyrocketing in many areas of life. For example, researchers are exploring the use of these scores to predict the onset of complex diseases, such as cardiovascular disease, diabetes and cancer. It has also been proposed that polygenic scores could be used to predict educational attainment (Lee et al., 2018), and social behaviors such as loneliness (Abdellaoui et al., 2018) and same-sex sexual behavior (Ganna et al., 2019). However, even when the association between a polygenic score and a certain phenotype is statistically significant, this does not always guarantee the polygenic score will have a strong predictive power.

Most phenotypes are the result of multiple genetic variations, which are found by screening the genome of populations and identifying which variants appear more frequently in individuals with a specific trait. Polygenic scores are then calculated for each person based on how many of these genetic variations are present in their genome. This score indicates how likely a person is to develop the phenotype of interest.

Studies using data gathered by the Avon Longitudinal Study of Parents and Children (ALSPAC) in the UK have identified various factors that can predict the educational performance of individual students, including cannabis and tobacco use, and month of birth (Wright et al., 2018; Odd et al., 2016; Stiby et al., 2015). However, it is unclear whether polygenic scores can predict student performance better than other information that is easier to obtain.

Now, in eLife, Tim Morris, Neil Davies and George Davey Smith from the University of Bristol report the results of a study in which they explored if polygenic scores could be used to predict the educational performance of 3,500 children from the ALSPAC cohort who were born in the early 1990s (Morris et al., 2020). The educational achievement of each student was determined by averaging test scores from national exams taken at 7 and 16 years of age. The team then compared these exam scores against both polygenic scores and other characteristics available to the school (such as age, sex, and Free School Meal status), and the education and socioeconomic position of the children’s parents.

Morris et al. found that although polygenic scores display some degree of predictive power, socioeconomic factors, such as parent education, are a better predictor for how well a child will perform in school. Moreover, earlier educational achievements were found to be the best indicator for educational performance: for example, the results of tests sat at age 14 can predict how well students will perform in tests at age 16. Therefore, polygenic scores are better at predicting earlier performances in school than later academic successes. However, the power of this prediction is still weaker than other, more easily measurable factors.

These differences in predictive performance are similar to what is seen in complex diseases: polygenic scores on their own are poor predictors and only minimally improve predictions made on the basis of other (readily available) data. Furthermore, just as early school grades predict later grades, early symptoms of a disease are an excellent indicator for how severe the condition may become (Meigs et al., 2008). This suggests that if major risk factors develop and influence the phenotype over time, predictions made before the emergence of these risk factors will be less informative.

Polygenic scores are always created using variables that we know are associated with the phenotype of interest, so they will always have some predictive power. Therefore, what we really want to know is whether this predictive power is high enough to be useful for practical applications. And to answer this question we need to know more about how the polygenic scores are intended to be used (Martens and Janssens, 2019).

Other studies on factors that influence the educational performance of the ALSPAC cohort did not use averaged test scores as a read-out of academic success. Instead they focused on how different factors predict the likelihood that a student would drop out of school, or finish secondary school with fewer than five C+ grades – the minimum requirement for most education and training courses after age 16.

If the aim of education policies is to get students to finish school with five or more C+ grades, then it is important to identify which students are most likely not to achieve this goal. These children can then be offered more teaching and a greater level of support. Knowing when these interventions should be introduced will inform at what age the education performance of a student needs to be predicted, and which predictors are already available. Therefore, if polygenic scores are going to inform education policy, it is important that future prediction studies are designed with the intended use in mind.
